# Dr. Foville on the Structure of the Brain

**Published:** 1840-07

**Authors:** 


### DR. FOVILLE ON THE STRUCTURE OF THE BRAIN, AND ON ITS RELATIONS TO THE SKULL.

Dr. Foville, already well known for his valuable researches on cerebral pathology, and also for the enquiries into his normal structure of the brain, has recently presented an important memoir on the latter subject to the French Academy, of which we are enabled, by the report upon it drawn up by MM. Bouillaud and Blandin, to furnish the following account. A more detailed analysis we shall hereafter give, when Dr. Foville's work comes before us for review.

The principal part of the memoir appears to be occupied with an enquiry into the respective course of the two layers of fibres, which Dr. Foville had demonstrated in 1825, and which have been since generally acknowledged to exist in the crura cerebri, the one *inferior* and *anterior*, continuous with the pyramids; the other *superior* and *posterior*, and specially connected with the posterior part of the medulla oblongata. These two layers may be traced forwards into the optic thalami and corpora striata, and thence were supposed to radiate to the different parts of the hemispheres. Dr. Foville has since devoted himself to ascertain the course of the fibres proceeding from the several fasciculi contained in the medulla oblongata, with much greater minuteness. According to his present statement, the *pyramidal* fibres, after passing through the optic thalami and corpora striata, radiate in two planes, which are entirely distributed to the convolutions forming the external and convex portion of the hemispheres. The fibres proceeding from the *posterior* part of the medulla oblongata also divide into two planes, which encircle the others in a remarkable manner; of these the *superior* makes its exit from the exterior of the corpus striatum and thalamus opticus, soon curves upwards and inwards, and constitutes the corpus callosum, the great commissure of the hemispheres. The *inferior* plane passes out, on the contrary, beneath the pyramidal tract, and gives origin to the optic and olfactory nerves, and then constitutes a white space, superior to the corpus striatum, interior to the fissure of Sylvius, posterior to the frontal lobe, and anterior and interior to the temporal lobe, which is perforated by a number of vascular foramina symmetrically disposed. According to Dr. Foville, this place is a centre from which proceed and in which terminate several sets of arciform fibres, which form circles enveloping the pyramidal portion of the crus, and terminating severally in the hemisphere. To this group belong the tænia semicircularis, and others hitherto undescribed. This part of the description is obscure in the report, from the brevity with which it is rendered; but the following may be regarded as the general results of M. Foville's investigations:

The cerebral convolutions form two distinct classes; one set crowning the summit of the fibres ascending from the anterior pyramids, and in relation, therefore, with the anterior roots of the spinal nerves; the others upon the course of the posterior fibres of the medulla, and also connected with the three cranial nerves of special sense. Hence, according to Dr. Foville, it is in the external and convex surfaces of the hemispheres that the motive influence is chiefly originated; whilst their plane surfaces, and the inferior part of the temporal lobe, minister to the sensory actions. It would also seem that the commissural fibres are entirely derived from the posterior fasciculi, and thus that the sensory nerves may maintain their connexion with both hemispheres, when the motor being connected only with one are paralyzed by an injury to it; and thus loss of motion in hemiplegia is much more common than loss of sensation. He is fully convinced that the fibrous portions of the brain, like the tissue of the nervous trunks, is to be regarded only as a *conductor*; and that the cortical substance is the *material substratum*, by the intervention of which the will directs the movements.

The reporters advert to the researches of M. Gerdy on the same subject, published some time ago, as corresponding in many particulars with those of Dr. Foville. Both seem to have arrived at the same general conclusions; and they differ only in particulars. The former has investigated most carefully the annular disposition of the fibres already adverted to; the latter has devoted his chief attention to the substantiation of the fact, most curious if true, that these fibrous circles proceed from and terminate in the posterior part of the medulla, and are thus a portion of the sensory tract; and that to this system of fibres the commissures belong.

We are disposed to feel much confidence in these statements, because we know Dr. Foville to be a most patient observer, and excellent anatomist, as well as a philosopher in the most enlarged sense of the term. Moreover, they fall in rather curiously with some views we formerly propounded, as to the parallelism between the cortical structure of the brain and the granular matter surrounding the terminations of the sensory nerves. (Vol. IX., p. 99.) We there contrasted motor or efferent* nerves, originating in the vascular plexus of the cortical substance, and having no free terminations in the muscles, but returning by a series of loops—with the sensory or afferent, which originate in the peripheral vascular plexus, and run towards the brain, where they were supposed to terminate. But the researches of Foville seem to show that they *do not* terminate there, but return by a series of loops in the cerebral substance, coming into relation with the cortical structure, on which they may be supposed to act, as the afferent fibres do with the muscular tissue.

The second part of Dr. Foville's memoir is occupied with some curious observations upon the relation between the osseous protuberances on the cranium and the *retreating* points of the brain beneath. Thus, he remarks, if we were to make an incision through the frontal eminence, perpendicular to its surface, and pursue this to some depth, we should arrive at the anterior cornea of the ventricle. In the same manner we should be conducted from the occipital protuberances to the posterior cornua; and from the parietal eminences to the large central cavity of the ventricles, in which the cornua meet: and that thus the form of the osseous covering is influenced by the condition of the ventricles to a great extent. He carries out this position in a very interesting manner; showing that, where the convolutions are large and the brain solid, the bony casing takes their form and impression; but, that where the ventricles have been distended with fluid, as in chronic hydrocephalus, they exercise an influence on the bony casing far greater than the convolutions, and the frontal, parietal, and occipital eminences are very large, whilst the impressions of the convolutions are faint. This fact, which many of our readers have doubtless remarked, has an important bearing on the general question as to the influence of the condition and development of the brain upon the size, form, &c., of the cranium. We shall look forward with much interest to the appearance of Dr. Foville's memoir.

---

### MEDICAL REFORM.

* The word *afferent,* at line twelve from bottom, in the page referred to, is a misprint for *efferent.*

